# Endovascular treatment of acute aortic isthmian ruptures: case study

**DOI:** 10.11604/pamj.2017.28.217.7531

**Published:** 2017-11-09

**Authors:** Mohamed Rida Ajaja, Amine Cheikh, El Mehdi Moutaouekkil, Mohcine Madani, Moha Arji, Amine El Hassani, Brahim Lakhal, Anas Slaoui

**Affiliations:** 1 Department of Cardiovascular Surgery, Cheikh Zaid Hospital, Rabat, Morocco; 2 Abulcasis University, Faculty of Medicine, Department of Pharmacy, Cheikh Zaid Hospital, Rabat, Morocco; 3 Cheikh Zaid Hospital, Department of Intensive Care, Rabat, Morocco; 4 Mohammed V University, Faculty of Medicine and Pharmacy, Department of Pediatric, Cheikh Zaid Hospital, Rabat, Morocco; 5 Mohammed V Univerity, Faculty of Medicine and Pharmacy, Department of Vascular Surgery, Rabat, Morocco; 6 Abulcasis University, Faculty of Health Sciences, Department of Cardiovascular Surgery, Cheikh Zaid Hospital, Rabat, Morocco

**Keywords:** Endovascular treatment, aortic isthmian ruptures, endovascular prosthesis

## Abstract

Traumatic rupture of the aortic isthmus is a rare lesion occurring in patients subjected to violent deceleration. Because of the forces involved, it is frequently associated with concomitant life-threatening injuries. The endovascular intervention has been described to be a feasible and efficient technique which may be proposed as a therapeutic option for patients with multiple traumas instead of delayed classical surgical repair after stabilization. We report the case of an adult who has had an accident with a traumatic rupture of the aortic isthmus associated with other lesions, our patient received endovascular treatment. The aortic prosthesis was imported from France and the surgery was done 3 days after the patient's admission. This procedure was performed for the first time in Morocco in our hospital with a multidisciplinary team. The prosthesis was implemented successfully and the result was very satisfactory on the heart level.

## Introduction

The aortic isthmus, located downstream of the left subclavian artery which is the most common location of acute traumatic rupture of the thoracic aorta [1]. Concerning practical part, the traumatic break of the aortic isthmus is a rare event arising in an almost constant context of polytrauma and the mortality-morbidity of which remains high [2, 3]. The diagnosis of break of the aortic isthmus widely benefited from the generalization of the not invasive exploration in the first rows of which appear the scanning and the transœsophagienne echocardiography. The recent advent of endovascular treatment disrupts conventional regimens of conventional open surgery; probably it should be evaluated on a larger number of casualties and the longer term to be able to specify the exact place [4].

The estimation of traumatic rupture of the thoracic aorta (RTA) presents around 13-20% of the injured died during an accident by a public road and is the second cause of death after traumatic brain injury. The aortic isthmus breaks are caused by violent trauma: road accidents in 75-90% of cases, falls from high places, violent direct shocks frontal and / or lateral burials. Their frequency increased with the speed of vehicles. The ejection of the vehicle doubles the risk of aortic rupture. Wearing a seat belt is effective against the consequences of frontal impacts but provides no protection against side impact. Male adults in their second and third decades are the usual target of violent trauma responsible for aortic ruptures.

Because of the violence of the trauma causal, it is unusual for the rupture of the isthmus to be isolated. Associated injuries affect all organs and their number is estimated to average four patients who die before they arrive at the hospital and two in survivors. Moreover, these associated lesions complicate the diagnosis, present problems at hierarchy of surgical procedures and influence the overall prognosis due to their own gravity and/or their potential aggravation by aortic clamping and heparin therapy (conventional surgery). Among the associated injuries they are the multiple vascular lesions whom urgently posing the most difficult problems [1,3, 5,6].

## Patient and observation

**Figure 1:**
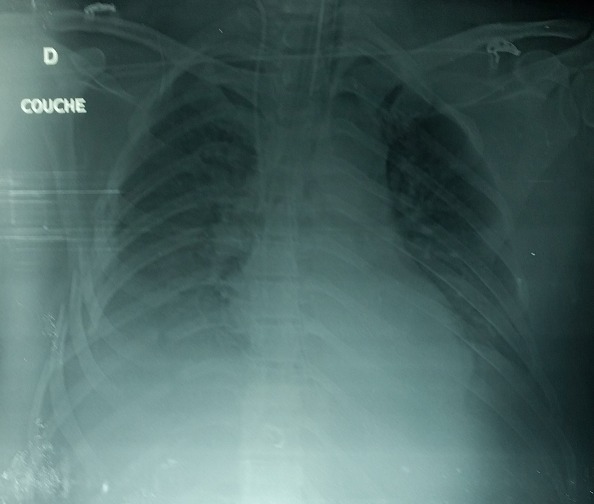
Odds fracture with left effusion

**Figure 2:**
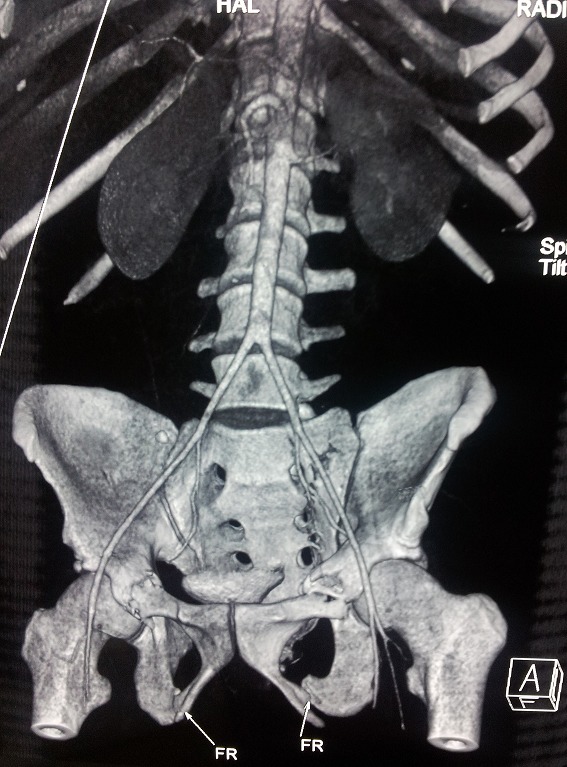
Non-displaced fracture of the pelvis

**Figure 3:**
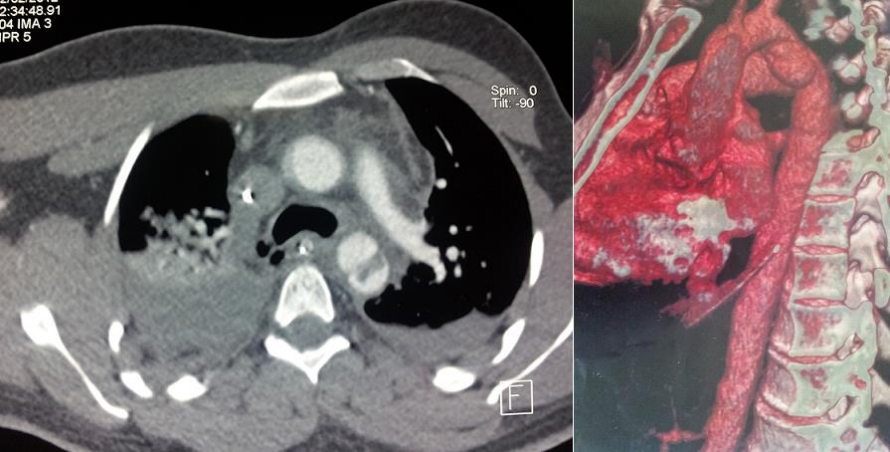
Rupture of the isthmus with right pleural effusion

**Figure 4:**
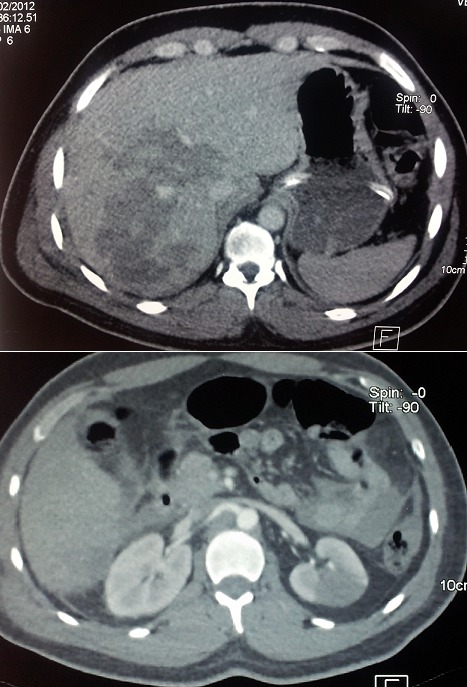
Abdominal CT scan revealed multiple liver and kidney contusions

Patient (32 years old) without significant medical history was victim of a road accident with a violent side impact. At admission, a patient was conscious (GCS 15) and stable hemodynamically. Cardiovascular examination is normal. The review pleuropulmonary exposes unduly bruises on the right side, and decreased breath sounds on auscultation right. The abdominal area, there is a sensitivity especially in the right upper quadrant. Therefore the remainder of the physical examination is unremarkable. The ECG was recorded in sinus rhythm regular, ventricular rate at 80 beats per minute. Without cardiac cavities hypertrophy, or repolarization disorder. The front pulmonary radiography shows staged costal fractures as one part of right lung contusions with chest flaps and a right pleural effusion (Figure 1) the radiography of the pelvis showed a non-displaced fracture of pelvis (Figure 2). Thoracic angio-CT scan indicated the aortic isthmus rupture with a pseudoaneurysm placed in the rupture. The thoracic aorta up, down and the abdominal aortic is without lesions (Figure 3). In addition, visceral lesions were indicated by angio-CT. The spleen and liver damage are for the foreground (Figure 4).

**Figure 5:**
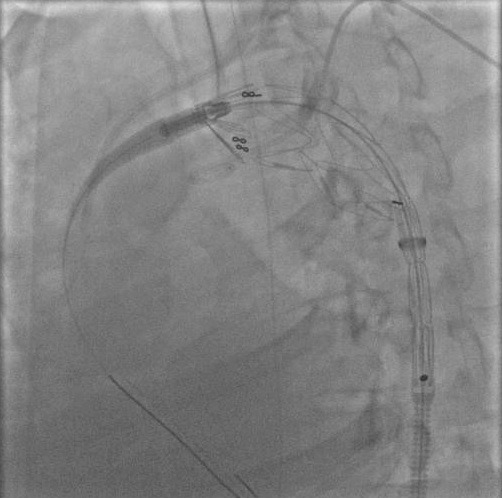
Introduction of the endovascular prosthesis

The rest of the lesional assessment was unremarkable. While laboratory tests proved: renal inadequacy due to rhabdomyolysis as well as the left renal bruise having required the use of hemodialysis. The hepatic and pancreatic tests were disrupted. In front of these numerous visceral hurts, an open-air surgical repair cannot conceive because of the incurred risks: horrifying hospital mortality, hemorrhagic risk, sees at first: worsening of the already precarious respiratory state, risk of paraplegia. The endovascular treatment (processing) by the introduction of endovascular prosthesis turns out the ideal solution in our case (Figure 5).

## Discussion

### Advantages and limits of endovascular treatment

The advantages of endovascular treatment with regard to the surgical treatment are multiple: 1) The intervention takes place in dorsal decubitus, what allows to avoid the postero-side thoracotomy among which the installation and the prolonged position are noxious at polytrauma more or less unstable patients. Note that in case of rough decompensation of an lesion the laparotomy as a matter of urgency remains possible; 2) It avoids the lung exclusion at patients with lungwort bruise and hypoxemia; 3) It avoids aortic clamping and prevents the increase in intracranial pressure at patients with traumatic brain injury; 4) The heparinisation is limited to 50 IU / kg in trauma patients at hemorrhagic risk; 5) The surgical aggression is lesser, a significantly shorter duration.

Several authors have introduced in recent years, a series of patients handled by endovascular way with good results in terms of post-operative mortality and occurrence of post-operative paraplegia. Amabile et al [7] present a series of 20 consecutive patients between 1998 and 2004; the first 11 ones handled in a surgical way (single clamping or Cardio-pulmonary bypass), while the last nine ones treated with endovascular way.

The operative mortality was 9.1% (1/11) for surgery, 0% for the endovascular group without paraplegia. The average follow-up of patients who underwent the stenting was 15.1 months (3 ~ 41 months). It wasn’t noted by deterioration of the stent and the exclusion of the isthmus lesion was acquired on the scanner to three months. One stent was required for each patient. Rousseau et al [8] present a series of 76 consecutive patients between 1981 and 2003; 29 patients were treated with stent with an average deadline after the trauma of 70 days (1-245 days) and a mean follow up of 46 months (13-90 months). No deaths and no paraplegia are to be regretted during the follow-up of these patients (21% of mortality vs 7% of paraplegia in the group of 28 patients operated as a matter of urgency and 0% of mortality with 0% paraplegia in the group of seven patients operated in a deferred way). Only an iliac break per procedure will oblige the realization of a vascular decking. The exclusion of the pseudoaneurysm was obtained in all patients, and it was not noted deterioration of the endoprosthesis. To our knowledge, there has not to date, no cases of paraplegia reported in the literature (French and English) after endovascular treatment of acute rupture of the aortic isthmus. However, these excellent results brought back in the literature, in terms of mortality, complication of spinal and good primary and mid-term results are qualified.

The limitations of endovascular treatment: 1) This raises the problem of the availability of emergency equipment and logistics. Several sizes of stents should be immediately or readily available. Fattori et al [9] and report the case of two patients treated in emergency thoracotomy due to unavailability of appropriate diameter stent; 2) Surgically, the surgical approach for stenting is common femoral artery. At the patients with arteritis, a surgical access of a common iliac artery sometimes has of the realized being increasing the aggressiveness of the procedure. Similarly, the possible occurrence of various complications is still possible with this technique: a broken iliac artery requiring bypass surgery during the procedure [8]. Another case of acute compression of the bronchus left origin with atelectasis homolateral lungwort is due according to the author to a rough increase of the pressure inside the thrombosed pseudoaneurysm frankly; 3) For several authors [8, 9] a healthy proximal neck is required downstream of the ostium of the left subclavian artery, in order to achieve the implementation of the stent. However, in several series, the ostium of the left subclavian artery was covered, sometimes completely without consequence [9].

The duration of follow-up of patients treated with endovascular way does not exceed a few years and the issue of long-term fate stents arises, especially as the population affected by this pathology is mostly young and some people of them wish to take back a professional or/and physical activity. Finally, the endovascular prosthesis exposes to a risk of secondary infection. The post-traumatic aortic ruptures are responsible from 10-15% of deaths after traffic accident. This is the second cause of death after traumatic brain injury. On the autopsy series, the incidence of traumatic rupture of the found aorta is quite variable. Strassman G found in 1947, 7000 autopsies performed between 1936 and 1942, 72 aortic traumatic ruptures that is approximately 1% among which 71% arisen during an accident of the public highway [10]. Another study conducted between 1961 and 1965, 42 of aortic isthmian ruptures on 1259 autopsies or 3.4% including 83% from the public highway accidents.

These post-traumatic breaks of the aorta are recognized in 90% of cases during accidents of the public highway and in 10% of cases during falls from a high place. These hurts are grave because 80% of the wounded persons die on the scene from accident and that only 20% of the victims will survive the initial trauma and will be transferred towards hospital. The number of patients victims of road accidents brought to the hospital and holders of post-traumatic aortic rupture is estimated at four thousand.

In the genesis of the rupture of the aortic isthmus several mechanisms have been implicated: 1) The hemodynamic factor during a thoracic trauma, the intra thoracic pressure is important. This increase of pressure and the parietal tension which result from it can favor the tear; 2) The mechanical factor: generally, the aortic breaks are secondary in an indirect mechanism; by deceleration in the axis of the body without chest trauma (accidents skydiving, plane, elevator, defenestration), deceleration and low compression of the thorax (the occupants of a car) and by antero-posterior compression; 3) The frequency of isthmic attacks is approximately 62%, with a significant proportion of lesions of the ascending aorta, the butt (24%) and the dawnward thoracic aorta (13.5%) [11]. Because of the violence of the trauma causal, it is unusual for the rupture of the isthmus is isolated. The associated injuries affect all the organs and their number is estimated to average 4 patients who die before their arrival to the hospital and to the two at survivors.

It may be: 1) Cranio-cerebral injuries: they are often the key to the forecast and therapeutic strategy. They can raise the problem of an urgent neurosurgical movement. The use of heparin in effective dose during surgical treatment can deteriorate brain damage such as bruising; 2) Pulmonary contusion: it is a lesion frequently observed during the breaks of the aortic isthmus, it is responsible for hypoxemia by variable severity; 3) Diaphragmatic ruptures: testify of a violent trauma and usually seen on the left (85-90%). Their frequencies in the literature vary from 1.8 to 7%; 4) Visceral lesions: they also belong to a deceleration mechanism and should be sought in case of hemoperitoneum. The hollow organ damage is rare. The spleen and liver damage are for the foreground (Figure 4); 5) Bone lesions: patient presenting a traumatic aortic rupture is often a polytrauma. He has multiple fractures femur, leg, pelvis, chest, skull, and spine.

The lesion severity score or ISS is a very important predictive and prognostic factor of morbidity and mortality. A score upper to 50 points is predictive of mortality superior to 50%. A score greater than 70 points is predictive of greater than 70% mortality. The multislice and spiral CT has become the gold standard; it is performed in emergency and first-line standard practice in cases of suspected rupture of the isthmus or systematically in case of polytrauma. Indeed, this test is realized easily and quickly. It allows the lesional assessment, not only of the thorax but also, and at the same time, the abdomen, head, spine (in particular cervical) frequently in a polytrauma patient.

In addition it allows a precise morphological assessment before endovascular treatment. It has a sensitivity of 100%, a specificity of 96% and a negative predictive value of 100% [12]. The transesophageal echocardiography (TEE) can be performed quickly, especially on a sedated patient in the intensive care unit or the operating room. The aortic isthmus is very well seen on transesophageal echocardiography. This examination allows besides estimating the myocardial function that can be altered in a context of chest trauma. TEE is a very specific examination (95-100%) and little sensitive (93%).

The isthmic aortic rupture remains a serious disease for which a high mortality persists. While few publications report a significant number of cases, there is a meta-analysis by Von Oppell [13] whose results are interesting; overall mortality was 32%, with a third before arrival in the operating room during surgery 7.8% and 13.5% postoperatively. Therefore endovascular treatment represents the treatment of choice at patients with multiple traumas, avoiding any significant heparinization and enabling the fast processing of associated lesions. The therapeutic management of isthmic aortic ruptures tends to change radically from the traditional surgical to endovascular treatment.

Endovascular techniques, less invasive and easier to implement at these trauma patient’s they have multiple benefits. Indeed, this treatment may be immediately offered, and this, whatever the associated lesions because it is minimally invasive, it doesn’t require a healthy vascular limited area; doesn’t contain an aortic clamping and requires only limited heparinization. The procedure is quick as simple and can be performed in the immediate waning processing a “priority injury” in the same operative session. Furthermore, its relative safety broadens the indications for older patients with comorbidities.

The main technical limits are at the moment, the small diameter of the thoracic aorta and a sharp curve of the aortic arch. A small caliber femoral arteries justifies using a primitive aortic or iliac access. The left subclavian artery is usually near the rupture zone, its cover, brought back in 26% of cases is generally well tolerated. Specific complications are represented primarily by the postoperative endoleaks reported in 5.3% of cases related to excessive oversizing or acute curve of the aortic arch.

The duration of follow-up of patients treated by endovascular does not exceed a few years and the issue of long-term future of endo-prosthesis arises, especially as the population affected by this disease is mostly young and some patients want to take a physical, occupational and/or sport.

## Conclusion

The traumatic rupture of the isthmus remains a serious disease and the management of which over the years has been greatly upset. If the treatment of traumatic isthmian lesions is using increasingly endovascular techniques, given their excellent results in terms of immediate morbidity and mortality, only the long-term results will confirm that attitude.

## Competing interests

The authors declare no competing interests.
